# The Impact of “Emergency-only” Hemodialysis on Hospital Cost and Resource Utilization

**DOI:** 10.5811/westjem.2022.11.58360

**Published:** 2023-02-24

**Authors:** Farina Shafqat, Shamie Das, Matthew A. Wheatley, Lauren Kasper, Sarah Johnson, Stephen R. Pitts, Michael A. Ross

**Affiliations:** * Emory University, Department of Emergency Medicine, Atlanta, Georgia; † Emory University, Department of Nephrology, Atlanta, Georgia

## Abstract

**Introduction:**

Undocumented immigrants are excluded from benefits that help compensate for scheduled outpatient hemodialysis (HD), compelling them to use emergency departments (ED) for HD. Consequently, these patients can receive “emergency-only” HD after presenting to the ED with critical illness due to untimely dialysis. Our objective was to describe the impact of emergency-only HD on hospital cost and resource utilization in a large academic health system that includes public and private hospitals.

**Methods:**

This retrospective observational study of health and accounting records took place at five teaching hospitals (one public, four private) over 24 consecutive months from January 2019 to December 2020. All patients had emergency and/or observation visits, renal failure codes (International Classification of Diseases, 10th Rev, Clinical Modification), emergency HD procedure codes, and an insurance status of “self-pay.” Primary outcomes included frequency of visits, total cost, and length of stay (LOS) in the observation unit. Secondary objectives included evaluating the variation in resource use between persons and comparing these metrics between the private and public hospitals.

**Results:**

A total of 15,682 emergency-only HD visits were made by 214 unique persons, for an average of 36.6 visits per person per year. The average cost per visit was $1,363, for an annual total cost of $10.7 million. The average LOS was 11.4 hours. This resulted in 89,027 observation-hours annually, or 3,709 observation-days. The public hospital dialyzed more patients compared to the private hospitals, especially due to repeat visits by the same persons.

**Conclusion:**

Health policies that limit hemodialysis of uninsured patients to the ED are associated with high healthcare costs and a misuse of limited ED and hospital resources.

## INTRODUCTION

Over 6.500 undocumented immigrants suffer from end-stage renal disease (ESRD) requiring renal replacement therapy, most commonly hemodialysis (HD), in the United States.^1^ These vulnerable patients lack access to standard three times weekly HD, do not qualify for Medicaid and Medicare dialysis benefits, and are excluded from provisions of the Affordable Care Act.^2^ Undocumented immigrants have the option to buy private insurance, but at a high cost. Many are unable to afford insurance, since 40% have annual incomes <$34,000 for a family of four or <$16,000 for an individual.^3^ Given these barriers, this patient population must resort to the emergency department (ED) for emergency-only HD.

Emergency-only HD is covered under the 1986 Emergency Medical Treatment and Labor Act (EMTALA), which requires EDs to stabilize emergency medical conditions regardless of the patient’s ability to pay. Emergency-only HD is provided when a patient presents to an emergency department (ED) and meets criteria for emergent or life-threatening conditions, such as hyperkalemia, uremia, volume overload, mental status changes, etc, due to untimely dialysis. Emergency-only HD has been associated with a 14-fold increase in mortality compared to standard outpatient HD.^4^ Undocumented immigrants must tolerate this risk as emergency-only HD is their only option to sustain life.

Limited data is available regarding the impact of these policies on the hospital cost and resource utilization regarding emergency-only HD in the state of Georgia. Therefore, our objective in this study was to describe the impact of emergency-only HD on hospital cost and resource utilization in a large academic health system in Atlanta, Georgia.

## METHODS

We conducted a cross-sectional analysis of electronic health records (EHR) and accounting records at five different teaching hospitals. We included a high-volume public hospital and four private hospitals providing care in the same large academic system. Inclusion criteria for the study were patients with an ED or observation unit visit over the two years from January 1, 2019–December 31, 2020 with either an *International Classification of Diseases, 10^th^ Rev, Clinical Modification* code I12.x or I13.x, or a Current Procedural Terminology code 82000002 for HD and an insurance status of “self-pay.” We excluded patients who were admitted to inpatient status. We obtained data from hospital EHR and from two separate accounting databases (Strata in the public hospital and EPSi in the private hospitals). The main objective was a simple description of the resource burden of emergency-only HD, including frequency of visits, total (direct and indirect) cost, and observation unit length of stay (LOS). In a secondary analysis, we evaluated the variation in resource use between persons and compared these metrics between the private and public hospitals.

We excluded 141 patients with observation unit stays of >48 hours, because they were likely miscoded hospitalized patients, as shown by correspondingly higher average cost. Statistical analysis included mean, median, sum, variance estimates, and differences in means. We used Stata Statistical Software Release 17 (StataCorp LLC, College Station, TX) for all calculations and production of all figures.

## RESULTS

During the 24-month study period there were 15,682 visits for emergency-only HD by persons without insurance, excluding HD visits that resulted in hospital admission. These visits were made by 214 unique persons, for an average of 36.6 visits per person per year. The average cost per visit was $1,363, for an annual total cost of $10.7 million. The average LOS per visit was 11.4 hours. This resulted in 89,027 observation-hours annually, or 3,709 observation-days. See Table 1 for a breakdown of metrics by public-vs-private hospital setting.

**Population Health Research Capsule**

What do we already know about this issue?

*Emergency hemodialysis (HD) is associated with a multiple fold increase in mortality and cost compared to standard three times weekly hemodialysis*.

What was the research question?

*What is the impact of emergency HD on hospital cost and resource use in an academic health system in Atlanta, Georgia?*

What was the major finding of the study?

*Average cost per emergency HD visit was $1,363, for an annual total cost of $10.7 million. Average length of stay per visit was 11.4 hours*.

How does this improve population health?

*This study highlights the cost and resource burden of emergency HD on the healthcare system and the need to seek solutions for providing standard outpatient HD*.

**Figure 1:**
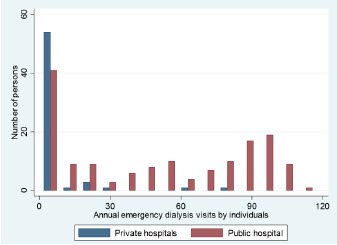
Distribution of annual visit frequency for emergency-only hemodialysis by individual persons, by hospital setting.

There was a high degree of variation in frequency of ED use for emergency-only HD between individuals, as shown in Figure 1, which plots the distribution of annual visit frequency per person by hospital setting and demonstrates the high-frequency users of the ED for HD. Not only was the overall frequency of emergency-only HD much lower in the private setting, the repeated use of emergency-only HD was also much lower in proportion, with only 16 persons receiving emergency-only HD more than once in the private hospital setting during the two-year study period.

**Figure 2:**
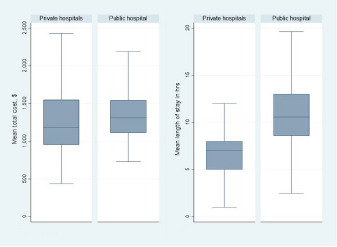
Box plots comparing cost and length of stay by hospital setting. Middle line is the median, box height is interquartile range, and whiskers represent Tukey minimum and maximum values.

The public hospital accounted for many more episodes of emergency-only HD for uninsured persons than the private hospitals, and much of this higher volume was due to repeat visits by the same persons. Since public hospital EDs allow much more recurrent HD by individuals, there was disproportionately greater impact by the few frequent visitors in the private EDs, as demonstrated in Table 2. The large differences in the apparent role of hospital setting (public vs. private) did not result in much difference in cost per visit, but there was a higher length of stay required in the public hospital. These differences are shown graphically in Figure 2. The difference in mean LOS was 4.0 hours (95% CI 3.6–4.4), and the difference in mean cost was $63 (95% CI 22–105).

## DISCUSSION

The results of this study reveal the high healthcare costs due to health policies that restrict HD access for uninsured patients to the ED, and these costs are likely to remain uncompensated. The highest burden of providing HD to these patients falls on the public hospital as shown in this study and similar studies performed in other states.^2^,^5^

The practice of requiring undocumented or uninsured patients to access HD services through EDs costs more and leads to worse patient outcomes.^4^ Patients who rely on emergency-only HD will often qualify for treatment fewer times than thrice weekly.^6^ This has been associated with increased inpatient hospital days and mortality.^5^ As previously discussed, admitted patients were excluded from this study and only emergency or observation visits were included. Hence, the high healthcare costs from this study do not include inpatient costs for this vulnerable population, and studies have shown that these patients are at increased risk of hospitalizations and intensive care unit stays.^4^ Therefore, the total costs of these health policies are much higher than those presented in this study.

Efforts have been made by other states to secure funding for undocumented immigrants to receive standard outpatient dialysis, and they have been shown to reduce cost, mortality, and hospital utilization.^5^,^7^ Approximately 13 states have expanded their emergency Medicaid provisions to reimburse standard outpatient dialysis.^8^,^9^ Currently, Georgia’s Emergency Medicaid does not cover outpatient dialysis. To determine possible cost savings if outpatient HD were to be provided to this population, we determined the cost per encounter for outpatient HD at a private HD center in Georgia. The average total expense for one outpatient HD encounter at this center was $309. This would lead to an estimated cost of $48,204 per year per patient for thrice-weekly dialysis. Furthermore, if all the encounters in this study took place in this outpatient setting, the total cost would equal $4,845,738, saving the health system $16,536,546.

This study highlights the healthcare cost and resource burden placed on EDs and the health system by policies restricting access to scheduled, outpatient HD for uninsured/undocumented patients in Georgia. It is imperative that policymakers find alternative solutions to provide regular outpatient HD to this vulnerable population in Georgia. Our team is reaching out to stakeholders to explore solutions and will use this study to help support the initiative.

## LIMITATIONS

This study does have some limitations. The insurance status of “self-pay” was used as a surrogate marker for undocumented patients, as the vast majority of undocumented immigrants with ESRD are uninsured.^10^ Chart review for high-frequency users from private and public hospitals was performed to determine the reason why these patients were uninsured. All high-frequency users at the public hospital were uninsured because they were undocumented at the time of the study. Half of the high-frequency users at the private hospitals were undocumented at the time of the study. Furthermore, this study was a retrospective review of cost accounting data, and the public and private hospitals had different data sources.

## CONCLUSION

Health policies that force undocumented/uninsured patients needing HD to visit the ED for emergency-only HD are associated with very high costs, misallocation of limited ED and hospital resources, and worse patient outcomes. Alternative solutions for providing regular outpatient dialysis to this vulnerable population are necessary.

## Figures and Tables

**Table 1:** Resource use by persons receiving emergency-only hemodialysis, by hospital setting.

	Total	Private	Public
Visits	15,682	566	15,116
Persons	214	61	153
Visits/person/year	36.6	4.6	49.4
Average cost ($)	1,363	1,302	1,366
Average LOS (hours)	11.4	7.5	11.5
Total annual cost (million $)	10.69	0.37	10.32
Total annual observation-days	3,709	88	3,621

*LOS*, length of stay.

**Table 2:** Impact of repeated emergency-only hemodialysis by the same patients, by hospital setting.

	Public hospital	Private hospital	
Most frequent 10% of visitors accounted for...	20%	89%	of visits
	22%	75%	of cost
	22%	71%	of observation-days
Most frequent 20% of visitors accounted for...	38%	99%	of visits
	39%	85%	of cost
	40%	80%	of observation-days
